# Understanding disadvantaged adolescents’ perception of health literacy through a systematic development of peer vignettes

**DOI:** 10.1186/s12889-021-10634-x

**Published:** 2021-03-25

**Authors:** Hannah R. Goss, Clare McDermott, Laura Hickey, Johann Issartel, Sarah Meegan, Janis Morrissey, Celine Murrin, Cameron Peers, Craig Smith, Ailbhe Spillane, Sarahjane Belton

**Affiliations:** 1 School of Health and Human Performance, Dublin City University, Dublin, Ireland; 2 Department of Sport and Health Sciences, Athlone Institute of Technology, Athlone, Ireland; 3 The Irish Heart Foundation, Dublin, Ireland; 4 School of Public Health Physiotherapy and Sports Science, University College Dublin, Dublin, Ireland

**Keywords:** Youth-centered, Participatory research, Intervention

## Abstract

**Background:**

Adolescence represents a crucial phase of life where health behaviours, attitudes and social determinants can have lasting impacts on health quality across the life course. Unhealthy behaviour in young people is generally more common in low socioeconomic groups. Nevertheless, all adolescents should have a fair opportunity to attain their full health potential. Health literacy is positioned as a potential mediating factor to improve health, but research regarding health literacy in adolescents and socially disadvantaged populations is limited. As part of Phase one of the Ophelia (OPtimising HEalth LIterAcy) framework, The purpose of this study was to explore the perceptions of socially disadvantaged Irish adolescents in relation to health literacy and related behaviours, and utilise this data to develop relevant vignettes.

**Methods:**

A convergent mixed method design was used to co-create the vignettes. Questionnaires were completed by 962 adolescents (males *n* = 553, females *n* = 409, Mean age = 13.97 ± 0.96 years) from five participating disadvantaged schools in Leinster, Ireland. Focus groups were also conducted in each school (*n* = 31). Results were synthesised using cluster and thematic analysis, to develop nine vignettes that represented typical male and female subgroups across the schools with varying health literacy profiles. These vignettes were then validated through triangular consensus with students, teachers, and researchers.

**Discussion:**

The co-creation process was a participatory methodology which promoted the engagement and autonomy of the young people involved in the project. The vignettes themselves provide an authentic and tangible description of the health issues and health literacy profiles of adolescents in this context. Application of these vignettes in workshops involving students and teachers, will enable meaningful engagement in the discussion of health literacy and health-related behaviours in Irish young people, and the potential co-designing of strategies to address health literacy in youth.

**Conclusion:**

As guided by the Ophelia framework, the use of authentic, interactive and participatory research methods, such as the co-creation of vignettes, is particularly important in groups that are underserved by traditional research methods. The approach used in this study could be adapted to other contexts to represent and understand stakeholders’ perceptions of health, with a view to explore, and ultimately improve, health literacy.

**Supplementary Information:**

The online version contains supplementary material available at 10.1186/s12889-021-10634-x.

## Background

Health literacy has been recognized as a potential intervening factor to reduce health disparities [1]. Health literacy is the ability of an individual to find, understand, appraise, remember and apply information to promote and maintain good health and wellbeing [2–4]. Accordingly, promoting and developing health literacy skills in early stages of life could contribute to reducing health inequalities caused by low health literacy levels [5].

Traditionally health literacy research and practice has neglected adolescents and focused on adult and clinical populations [5, 6]. There is evidently a need to explore, authentically represent and understand the perceptions of health in other contexts, with a view to improving health literacy [5, 7]. Identifying and responding to the specific needs of a target group in a relevant and meaningful way is a complex task. Participatory approaches pose a potential methodological solution as they suggest using a range of stakeholders to enable the co-design and co-development of interventions that understand and appreciate local problems, local needs and ultimately, arrive at local solutions [8]. This is particularly important when working with socially disadvantaged groups, where it has been suggested the research needs to operate via community partnerships [9], to be accepted, adopted and sustained [10].

The Ophelia (OPtimising HEalth LIterAcy) framework has been developed to specifically guide the co-design of health literacy interventions [11, 12]. The framework details three key phases of intervention development to enable participation, community focus, equity and sustainability [4]. The current paper details part of Phase One of the Irish Heart Foundation (IHF) Schools Health Literacy project, which is a registered World Health Organisation (WHO) National Health Literacy Demonstration Project, following this framework, working to co-produce a school-based health literacy intervention. The initial stages of The Ophelia framework proposes the co-creation and use of robustly, locally-developed vignettes [11–13]. These vignettes are short stories which enable stakeholders to engage in conversations around typically high, average, or low health literacy individuals in their community [12]. This is a pragmatic realist methodology, which provides an empowering and equitable way to gain rich insight and understanding into factors related to health literacy [11] . Vignette methodology has been used for many years to explore attitudes, values, norms and perceptions of health issues and other potentially sensitive topics [14–17]. They enable active and controlled discussion, particularly with young people, as they allow participants to differentiate from themselves, discuss their opinions, and identify in a non-threatening manner [14, 16]. Previous research has indicated that careful vignette development and interpretation is essential to ensure that they are relevant, realistic, and engaging for participants and that responses are understood as participants shift between speaking about themselves and the character [16, 17].

Crucially, the Ophelia framework develops vignettes in a participatory approach which actively seeks local wisdom [11, 13]. To the best of our knowledge, this approach has not been demonstrated in an underserved adolescent population in relation to health literacy. In the Republic of Ireland, the Delivering Equality of Opportunities in Schools (DEIS) action plan aims to address educational disadvantage [18]. For international consistency throughout this paper we will use the term ‘disadvantaged schools’ instead of DEIS. Disadvantaged schools are identified based on the socio-economic demographic data of pupil cohorts, and schools subsequently receive support such as access to grants, school meals, school completion programmes and home school liaison officer and guidance counsellor posts. Despite the introduction of the scheme in 2005, there remain substantial and significant gaps in school performance, retention, and in medical card possession between disadvantaged and non-disadvantaged school pupils [18, 19]. Longitudinal data also shows that pupils in disadvantaged schools tend to have higher levels of overweight and obesity and the gap becomes wider as children get older [20]. As a result, programmes to target health related areas can potentially have the greatest impact within disadvantaged schools.

The following paper details the development of a series of vignettes for use in disadvantaged post-primary schools in Leinster, Ireland. This study is part of the Irish Heart Foundation’s (IHF) Schools Health Literacy project, which is a registered World Health Organisation (WHO) National Health Literacy Demonstration Project and the first such project to focus on primary prevention of cardiovascular disease and young people. The overall aim of this project is to improve the health literacy and subsequent health outcomes of adolescents in Ireland. It should be noted that this study was conducted in 2019, before the outbreak of COVID-19 (Coronavirus- 2019).

## Methods

### Participants

Five disadvantaged schools, who had existing relationships with the IHF through their schools programming, were contacted to invite expressions of interest for involvement in the study. Schools were invited on the basis of ensuring a mix of single gender and mixed gender schools, urban and rural schools. Upon Principal consent to school involvement, all students in the first 3 years of schooling (ages 12–16 years old, commonly referred to as the ‘Junior Cycle’) in these five schools were invited to take part. In total, nine hundred and sixty-two adolescents (mean age of 13.97 ± 0.96, females *n* = 409; four urban schools, one rural school; two mixed schools, one all male school, and one all female school) provided informed consent and were present on the day of data collection, and subsequently completed the questionnaire. A subsample of 31 participants from across the five schools were selected by the schools to take part in a focus group. One focus group was conducted in each school with two students from each of first, second and third year participating; with the exception of one school that had a group of seven students participate in the focus group from across these year groups. Dublin City University Ethical approval was granted for this study by the institutional ethics committee [DCUREC/2019/053]. School and parental informed consents and participant assent were obtained prior to participation.

### Procedure

A convergent mixed methods design was used in the current study. This design was adopted as it was most appropriate to address the research question [21, 22]. Quantitative and qualitative data was collected, and given the pragmatic nature of working with schools, in practise questionnaires were conducted prior to focus groups [22]. After the completion of data collection, initial data analysis was conducted in parallel, with findings from both questionnaires and focus groups ultimately synthesised and integrated together resulting in the co-production of a series of vignettes.

#### Questionnaire

In a standard classroom, during normal school time, participants completed questionnaires on tablet devices (Microsoft Surface Go) using offline software ‘Survey anyplace’ with a unique assigned ID number. Prior to starting the questionnaire, the lead researcher explained the purpose of the study, provided instructions on how to complete the questionnaire and encouraged participants to answer the questions honestly. A standardized protocol for questionnaire administration was used throughout. Participants were encouraged to take time to reflect on their answers and to be as honest as possible at all times. The maximum ratio of participant to researcher was 6:1.

#### Focus group

The five focus group interviews were conducted in the five schools following the questionnaire data collection over a three-week period in May 2019. Focus groups were carried out in a classroom and were 40 min on average. One researcher led the session, with one research team member observing and taking notes. At the start of the focus group, the researcher reminded participants that they could withdraw from the focus group at any stage, and that all recordings would remain confidential. All focus groups were audio-recorded using a digital dictaphone and transcribed verbatim.

### Material

#### Questionnaire

At the time of conducting this research there was no existing validated adolescent health literacy questionnaire for use in a non-clinical, adolescent population, readily available in English [23]. As a result, and in line with the Ophelia framework, which suggests that the operationalisation of health literacy should be flexible and responsive to the contextual needs [12], the questionnaire used in this study was developed specifically to meet the needs of the current study (please see supplementary information). The questionnaire was developed based on different sources including 10 items from the Health Literacy Questionnaire (HLQ) developed and administered among adults [24], 13 items from a questionnaire used for 11–14 year olds in the Healthy Start to Life Education for Adolescents Project [25], the PACE + two-item questionnaire to measure physical activity levels [26], along with four demographic questions, and 38 additional items, derived by the research team through consideration of relevant domains and items of the HLQ that were not included in the above, but were considered important to try to interrogate. The majority of these scales used Likert-type response options (e.g. 1 = Strongly Disagree, 2 = Disagree, 3 = Agree, 4 = Strongly Agree). The questionnaire was comprised of 67 questions in total, and on average took 11 min and 50 s to complete.

#### Focus groups

The purpose of the focus groups was to identify and explore perceptions and understanding of health literacy in adolescents in disadvantaged Irish schools. This rich, contextual information was sought to provide in-depth insight into the perceptions of young people in this project. With this in mind, a semi-structured focus-group guide, using questions designed by the research team who had specific expertise in qualitative research design, was developed. This included questions to consider understanding (e.g. what is your understanding of the term health literacy?), awareness (e.g. do you understand what you need to do for your health based in the information you find?), strengths (e.g. are there things you do regularly to make yourself healthy?), needs (e.g. is there somebody you can trust to understand and support your questions about health?) and issues in relation to health literacy and health throughout the school communities. Questions were piloted with two groups of similarly aged students (*n* = 16) from a Youth Advisory Panel recruited by the Irish Heart Foundation. The wording and ordering of questions was refined based on this pilot.

### Data processing and analysis

IBM SPSS Statistics 24.0 was used for analysis of the questionnaire data. Data were entered into SPSS and standardised to a scale of 0–1 for each variable. Only participants who had full data available for all variables were included in analysis. Principal component analysis (PCA) (with a varimax rotation) was used to identify and compute composite scores for the potential factors underlying the adapted health literacy questionnaire, in an aim to reduce the number of items into smaller components or factors by exploring interrelationships among the data set. To test if the data set was adequate for factor analysis, a measure of sampling adequacy (MSA) of Kaiser–Meyer–Olkin was applied. A minimum eigenvalue of 1.0 was used to accept a factor as statistically meaningful. Catell’s scree test was applied, and where a clear break was identified in the plot the factors above the break were retained. A coefficient of .3 or above was considered an important factor loading. The results of the PCA were used to guide categorisation of the items in the questionnaire into factors, where a two-step cluster analysis procedure was subsequently used to explore whether subgroups could be identified based on the initial factors developed from the PCA analysis, to form potential health literacy profiles of groups of individuals within this context [27, 28]. First, Ward’s hierarchical clustering method was conducted to obtain initial cluster groupings [29, 30]. Ward’s minimum variance method tends to derive more equally sized groups. In addition, squared Euclidean distance was used to measure the distance between the individual observations on the clustering variables.

Focus group transcripts were analysed using thematic analysis [31, 32]. This process initially required the reading of the individual transcript to assign broad thematic codes which was initially completed independently by two researchers. These broad codes were then subsequently organised into higher and lower order themes of significance and importance in relation to student’s perception of health and relative importance. Two researchers then critically reflected their engagement with the analysis, and cross-examined the data providing opportunity to explore, challenge and extend interpretations within the data [32].

The output from the qualitative and quantitative analyses were then synthesised. Data from the focus groups was used to interpret the cluster-profiles and to subsequently to generate several vignettes of the ‘typical’ types of students in this context, with four authors responsible for the initial drafting of vignettes (CMD, CS, HG, LH). Themes, quotations and contextual information derived from the qualitative analysis were used to illustrate characters across a range of health literacy levels and demographic descriptors (such as gender and age) which were identified by the cluster-profiles [12, 13]. The vignettes sought to represent a tangible, valid and authentic description of the health literacy needs of a comprehensive range of target students. A triangular consensus procedure was used, whereby vignettes were discussed between researchers, students, and teachers. These validation checks were conducted to ensure the trustworthiness and credibility of the developed vignettes. Throughout this process, wording and descriptive characteristics were altered until agreement was reached.

## Results

### Questionnaire

Principal Components Analysis resulted in eight factors being identified from the questionnaire data, with three to ten items included per factor. All items retained in each factor had primary loadings greater than 0.351. The research team generated factor labels for each factor; Lifestyle behaviors (9 items, load range 0.41–0.819), Information on risky behaviors (5 items, load range 0.351–0.786), Information on positive health behaviors (5 items, load range 0.472–0.7), Health information from media sources (10 items, load range 0.423–0.704), Social support (5 items, load range 0.359–0.707), Understanding health information (3 items, load range 0.651–0.812), Effect of lifestyle on health (6 items, load range 0.665–0.755), and Appraisal of health information (3 items, load range 0.437–0.720).

The eight identified factors were standardized to Z-scores to ensure equal contribution of variables in the cluster analysis. Using the Mahalanobis distance measure, multivariate outliers were also identified and deleted (*n* = 14), leaving a final sample of 941 participants. The number of cluster-profiles was selected based on the rescaled distances evident in the hierarchical cluster dendrograms, the percentage change in agglomeration coefficients at each step of the cluster analysis, and theoretical considerations (Hair & Black, 2000), which resulted in six cluster solutions. In the second stage, to refine the initial cluster solution, the cluster means from the hierarchical analysis were independently analyzed through a non-hierarchical k-means cluster analysis. Analysis produced the findings presented below in Table 1. A brief descriptive overview of each of the cluster-profiles was then developed (for example, see Table 2), prior to integration of the quantitative data with the richer qualitative data to generate the Vignettes (for the full series of vignettes, please see Supplementary File 3).

**Table 1:** Mean scores for Factors across Six Clusters

	Cluster 1	Cluster 2	Cluster 3	Cluster 4	Cluster 5	Cluster 6
	*N* = 201 (21%) 134 boys 67 girls	*N* = 119 (13%) 64 boys 55 girls	*N* = 187 (20%) 88 boys 99 girls	*N* = 91 (10%) 44 boys 47 girls	*N* = 188 (20%) 109 boys 79 girls	*N* = 155 (16%) 100 boys 55 girls
**Generated factor labels**						
Lifestyle behaviours	7.15	7.49	5.81	5.12	6.07	6.32
Information on risky behaviours	3.59	4.46	3.35	3.14	3.66	4.41
Information on positive behaviours	3.75	4.44	3.46	2.91	3.48	4.09
Health information from media	4.52	5.66	5.55	4.35	4.08	4.36
Social support	3.96	4.24	3.37	2.85	3.37	3.51
Understanding health information	2.23	2.51	2.12	1.64	2.14	2.48
Effect of lifestyle on health	5.24	5.29	4.73	3.76	4.25	4.96
Appraisal of health information	1.76	2.28	1.96	1.32	1.47	2.06

**Table 2:** Example of one vignette derived from the focus group data and cluster analysis

Lifestyle behaviors	Information on risky behaviours	Information on positive behaviours	4. Health Information from media	Social support	Understanding health information	Effect of lifestyle on health	Appraisal of health information
5.81	3.35	3.46	5.55	3.37	2.12	4.73	1.96
*Brief Descriptive overview from cluster analysis:* Cluster 3 included 187 students and was evenly distributed for gender. Scores for lifestyle behaviours, information on risky behaviours, information on positive behaviours and understanding health information were second lowest of all clusters. Scores for information from media sources were second highest of all clusters. Physical activity levels among participants in this cluster were also second lowest of all clusters with an average of 60 min of physical activity on 3.47 days per week.							
*Final Vignette (derived from qualitative data):* Emma is 14. Emma believes that if you want to be healthy and happy with yourself, you need friends to talk to. Her favourite thing to do is go out with the girls. They love going for food and looking around the shops. It’s good because without even realizing it, they actually walk a lot. Emma loves it when all her friends come around to her house because her Mam always order Dominos for them, and then they can go to McDonalds for a McFlurry after. Emma knows that if your parents are working all day and they’re coming home late, they’re wrecked so they don’t always have time to cook. She doesn’t mind, she loves pizza and ice cream. She loved the chipper around the corner where they used to live, but they had to move house and her Dad won’t drive to get it after he’s had a drink in the evening. Emma is addicted to her phone. She follows every fashion and make up influencer. She loves trying out the videos and doing her make-up. Sometimes she thinks that she’s overweight compared to other people. It takes Emma ages to get ready for school in the morning. Then after school, even if it’s just going to the park, it takes her even longer to get changed and choose what to wear. Emma used to play sport but stopped because her friends stopped going. She used to do dance but the classes were by her old house so she can’t go anymore. She still has to do PE in school, but she tries to forget her gear at least every second week. She doesn’t like having to get changed in front of everyone. Someone said something about what she was wearing and now it just feels like they’re staring at her every time she gets changed.							

### Focus group

The focus groups identified three higher order themes in relation to adolescents’ perceptions of health issues: i) The importance of mental health, ii) Sources and appraisal of health information and, iii) Adolescents perception of what constitutes health.

#### The importance of mental health

The importance of mental health was apparent throughout all of the focus groups. There were three lower order themes identified from the data, the first of which encompassed a view held by the majority of adolescents; that *mental health was more important than physical health for overall wellbeing*. Many felt that mental health was a more important contributor than physical health to leading a healthy life. Participant’s felt that poor physical health was not necessarily synonymous with poor mental health “You could be the most healthy mental person or have the best mental health and you could be the fattest guy on the planet”.

The second lower order theme was the issue of how *mental health stigma* is still prevalent, despite progress in mental health awareness. For example, “there is a lot of stigma around for mental health”, with another participant adding “they feel they can’t talk about it to anyone”.

The third lower order theme centred on *participants’ awareness of behaviours that maintain or compound mental health*. Adolescents displayed awareness for the need to maintain good mental health when some participants spoke of behaviours and activities that young people practice in an attempt to consciously look after their mental health. Some participants felt that “if you socialise a lot you are probably more healthy”. Others spoke of how mental health is starting to be discussed more openly “… there is this thing going around where it is OK not to be OK”. Additionally, adolescents emphasized that “schools and children are being taught that it is OK to be different”.

While the majority of adolescents spoke about health-promoting behaviours, a number of participants mentioned poor mental health behaviours that were linked to family and school issues, which led to a stressful environment. The school issues described by participants were often linked to a clash in home and school expectations. At school, participants felt teachers were encouraging them to study and strive to further their education, when in reality, the students were experiencing quite the opposite from home, leading to feelings of stress and anxiety.

Adolescents described a number of maladaptive coping strategies, including “locking yourself in a room and being away from society” and “self-harming” as a means to cope with problems encountered both at home and in school. Most of the negative mental health behaviours were linked to adolescents striving to fit in and conform to social norms, as well as the presence of bullying and cyber-bullying. Social media appeared to add huge pressures to adolescent participants, where they felt it was crucial to document each aspect of their lives online for others to see, for example “Snapchat and Instagram and all and you’re thinking, oh, if I don’t put this picture up everyone is going to be like, oh, she isn’t doing anything with her life, do you know what I mean, like oh she’s so boring and stuff like that”.

#### Sources and appraisal of health information

The second higher order theme from the data described how adolescents attain information about health and how they process and appraise it. The first lower order theme identified was *the appraisal of external information sources*.

*Sources of health information* varied greatly among participants, but the main sources of such information included parents, friends, family, the school, and the media. Adolescents described that some school subjects, including home economics, social and personal health education (SPHE) and physical education were important sources for health information, in addition to the importance of social media; “On snapchat there are little ads popping up after someone’s story just saying, oh, why don’t you go for a walk or something”.

Not only did the source of information vary between participants, but the trust of these sources created debate among adolescents. It was apparent that depending on personal experiences, adolescents placed their trust in different people and places. There were major differences between participants in trusting information from their parents, with one participant adding “your parents might not know much about it”. Another participant commented they would trust their “football manager, I don’t trust my family”. There were also large variations in trust for school and teachers, with some participants agreeing to trust school “because if you think about it, everybody here has been to college” with others arguing “all the teachers just tell you different things and then some of them are just saying it so you will be quiet and stop asking questions”.

This linked to the second lower order theme *self-appraisal* referred to the reliance on oneself to attain and appraise information. A proportion of participants claimed they did not trust any source of information and strived to find information for themselves. Participants spoke of their lack of trust across various sources including school, parents and the internet. These participants made comments including “don’t trust any of them”, “there isn’t much information you can trust” and “everything is fake nowadays”.

#### Adolescents perception of what constitutes health

Within the higher order theme of *adolescent’s perception of what constitutes health*, there were three lower order themes identified. The *absence of negative thoughts* or the notion that to have negative thoughts was abnormal, the *blurred boundaries* that are apparent among adolescents between healthy and unhealthy behaviours, and *body image*, which was particularly apparent among female participants.

As discussed previously, mental health was one of the biggest contributors to *what constitutes health* according to adolescents and has been discussed in theme one. An interesting finding from the focus group interviews was that for some participants their perception of being healthy was to have only positive experiences and that if one had a negative thought or a negative experience in any domain of health then they were not classed as healthy. This was particularly referenced regarding negative thoughts. It was apparent that adolescents in this study were now so focused on the importance of positive mental health and practicing behaviours to enhance this positive mental health, that they felt it was not normal to experience any negative thoughts or feelings. When asked what it meant to be unhealthy one participant responded “to have negative feelings or anger or sadness” and another said “avoid any negative thoughts” and another stated that to be unwell was to “dwell on bad things that have happened in the past”.

Some *blurred boundaries* were apparent between what constitutes healthy and unhealthy behaviours, and this was framed as a second lower order theme for adolescent’s perception of what constitutes health. For the most part, adolescents understood that alcohol, smoking, vaping, drug consumption, inactivity and “fatty foods” all fell under the unhealthy category and that physical activity and consumption of healthy food were healthy behaviours. Examples of comments that displayed an understanding of healthy and unhealthy behaviours included some answers to the question, ‘what does it mean to be healthy?’ “to eat healthy and being active”, “getting enough sleep”, “more outdoor activity instead of like more screen time”, “getting on well with your friends”.

In, response to ‘what does it mean to be unhealthy’ for the most part, participants showed some understanding of unhealthy behaviours “if you’re smoking”, “not going to school”, “not fit enough”, “on drugs like people on drugs don’t feed themselves or nothing” and “drinking really big amounts like every day like waking up drinking and going to sleep drinking and things like that”. It is worth noting that there were some comments made by participants that displayed a normalization of unhealthy behaviours or perhaps a lack of understanding of the attributes to health. This ranged across the various behaviours associated with health. One participant discussed “physical activity is very optional” and a classmate responded, “yeah it’s kind of like some people don’t need physical activity” while another commented “… it varies from person to person”.

The normalization of behaviours was also discussed “I’d generally associate it (alcohol) with being unhealthy but in today’s society it’s normalized” and further comment about drinking alcohol among their age group suggested “have a bottle and don’t drink the whole crate of them” insinuating that there was no problem in having some alcohol at the ages between 12 and 16, but that the problem only arose when large quantities were consumed. With reference to smoking participants discussed that more second year students (ages 13–14) than third years (ages 15–16) smoked, because they felt that third year brought with it a sense of responsibility as, in Ireland, students carry out state exams in third year; “the junior (exam) is next year so let’s go all wild while we can”. In relation to cannabis, students spoke of medical use, for example, with cancer patients “yeah if it’s for medical purposes its good then” and with a lack of reference for the detrimental effects of such a drug.

The third lower order theme within this theme was the concept of *body image* which played an important role in defining health for adolescents; particularly for female participants, it was a common conception that body image portrayed health. When asked what does it mean to be healthy some responses reflected body image including “skinny”, “that your body is good” and “being comfortable in who you are” as well as when asked what it means to be unhealthy often the responses included “obese” and “overweight”. These comments reflect that young people perceive health to be directly equated to the physical appearance of a person, as opposed to holistic physical, social and mental wellbeing. It was also apparent that negative *body image* for both themselves and others was extremely common among adolescents, with a number of comments relating to judging themselves and other on their appearance” judging other people by their looks and their sizes”.

### Vignettes

In line with previous research following the Ophelia framework, clusters were made relevant and user friendly through the writing of vignettes [8, 12]. These short narratives illustrated how a typical person within each cluster might be living with that health literacy profile. Crucially, the current study synthesised the quantitative data with contextual insight gleaned from the focus groups to create accurate and authentic descriptions of typical students within this community considered to have high, average and low levels of health literacy.

Initially, eight vignettes, considering the cluster profiles, qualitative insights and a variety of demographic descriptors (gender and age) were developed. This included using explicit phrases identified within focus groups. All vignettes were written to a suitable Flesch-Kincaid Reading score for the age range of participants within the project. The vignettes were then presented to both the wider research and project teams respectively, who have extensive experience of working with participants in this context, to ensure accuracy of the interpretation and readability.

A validation process was then undertaken. The vignettes were presented to representatively similar students (*n* = 16) and teachers (*n* = 3) from in follow-up interviews and focus groups. Discussions started broadly, asking if participants ‘recognised’ this person within their school communities, before going into detail regarding word choice and phrasing, clarifying the length and readability of the vignettes, and finally, checking if there were any health issues they felt were important in their context, but not represented within the vignettes. Based on this feedback, a ninth vignette was created, depicting a girl with average health literacy who had a good understanding of risky behaviours, but did not apply their knowledge and understanding of health information. Contextually, this was characterised by students and teachers as a quiet, younger female, who was not physically active outside of school and who was beginning to display awareness of body image issues. This final vignette was again presented to a different group of students and teachers for refinement.

This rigorous approach was undertaken to develop contextually rich and empirically grounded vignettes [4, 8, 12]. This highlights the pragmatic approach needed to balance evidence-based considerations with real-world implications. Initial feedback from the validation process suggests that these vignettes will allow a range of potentially sensitive health related topics to be discussed. An example of one health literacy profile is shown in Table 2 (for further vignette examples, please see supplementary information).

## Discussion

This paper sought to describe part of Phase One of the IHF’s Schools Health Literacy project, which is a registered WHO National Health Literacy Demonstration Project, following the Ophelia framework, which resulted in the co-creation of a series of vignettes. This process undertook a participatory, co-design methodology, which promoted the engagement and autonomy of the young people and teachers from the five schools participating in the ongoing project.

Adolescence is a crucial stage in life for emotional, social and cognitive development, and a critical period for the formation of health-related behaviours [6, 33]. Adolescence is also a period when health behaviours and social determinants, such as the ability to stay in education, can have lasting impacts on health equity across the life course [34]. Unhealthy behaviour in young people is generally more common in low socioeconomic groups [35–39]. The Global Strategy for Women’s, Children’s and Adolescents’ Health (2016–2030) and the Global Accelerated Action for the Health of Adolescents (AA-HA!: guidance to support country implementation) stress that all adolescents should have a fair opportunity to attain their full health potential, and should not be disadvantaged from attaining that potential [40, 41].

There has been a move in recent years in many countries worldwide from a focus on health literacy in the medical or health care settings to a much broader consideration, including schools and young people [42]. Vamos et al. [42] recognise the education sector as a critical domain towards achieving health and wellness goals for individuals across the life span. The authors propose an ‘education for health literacy’ perspective, which suggests health literacy is a key outcome of health education [42]. In Ireland, recent curriculum reform has seen the introduction of ‘Wellbeing’ as a programme area for the first time [43]_._ This new programme of study combines three previously existing subject areas, Physical Education (PE), Social Personal and Health Education (SPHE), and Civic Social and Personal Education (CSPE), and became a compulsory programme of study for all students, in all post primary Irish schools from September 2017 [44]. Critically within this curriculum reform, Wellbeing is recognised not only as a programme of study, but also a whole school endeavour for Junior Cycle (first 3 years of post-primary education, the population at the centre of this article) education in Ireland, with the four areas of Culture, Curriculum, Relationships, and Policy and Planning identified as the key aspects for schools [44].

The documentation produced for schools on this new overarching Wellbeing area [44] provides solid guidance and initial structure, which has been subsequently supplemented by the work of the Junior Cycle Team (a group set up by the Department of Education and Skills to provide CPD support to schools and teachers in enacting curriculum reform). Wellbeing guidelines are not prescriptive however and allow for schools to interpret and enact Wellbeing in a way that best meets the needs of their students. Schools are encouraged to take a whole school approach to developing wellbeing programmes and initiatives which are collaborative, consultative, responsive to students needs and context, adaptable to new and emerging circumstances, and linked to whole school planning (Junior Cycle for Teachers, 2017). Lund and Tannehill [45] speak to the importance of having clear outcomes in mind for any curriculum development, with curriculum being that broad whole school consideration, rather than subject oriented and confined to the classroom. Health literacy as an outcome of Wellbeing, across a range of areas of study (such as PE and SPHE), but also across the other three areas of Culture, Relationships, and Policy and Planning, needs little justification. Lund and Tannehill [45] further emphasise the question of ‘what do students need to know and be able to do at this point in their lives’ as an essential starting point when developing a curriculum area (be it at a subject of a whole school level). This aligns also with the focus emphasised by the NCCA in the new Wellbeing programme area; ‘*When schools have a strong focus on taking care of the needs of all students, then those children who are vulnerable or experiencing difficulties also benefit*’ (pp28, 40). Critical to understanding what the needs of students may be with respect to health literacy and wellbeing, is considering the voice of students and teachers from the outset.

The salutogenic approach taken in Ophelia [11, 12] to the co-design of interventions to promote health literacy, offers a firm framework to the development of health literacy interventions in health care settings and beyond. The current study, in purposively aligning with the Ophelia principles [11, 12], represents the first stage of the development a health literacy intervention for students attending Junior Cycle education in disadvantaged schools in Ireland. Set against the backdrop of the curriculum reform outlined in the previous paragraph, the study sought to identify the current context for health literacy in adolescents in schools, and through the collation of this information, the subsequent development of vignettes that can be used to stimulate meaningful co-design with participants. As such, and in line with [46], the vignettes developed within this study are contextually specific, providing rich insight into the experiences, challenges and perceptions of young people in disadvantaged post-primary schools in Leinster, Ireland. While the descriptions themselves by definition are context specific and may not be directly applicable to other populations and contexts, the methodologies utilised nonetheless provide useful detail, and expands the evidence base in this area. The mixed methodology and subsequent analysis has allowed for the development of rich, authentic and tangible descriptions of the health issues faced by adolescents in disadvantaged Irish schools, grounded by the rigorous and comprehensive health literacy cluster profiles generated via the survey data.

The practical implications of the work outlined in this paper, which has been initiated taking a pragmatic approach to health literacy intervention development are that it offers a solid foundation for the schools in question to explore how health literacy may be targeted, consistent with the approach advocated by the NCCA and the JCT for starting to integrate the area of Wellbeing within their schools. The planned use of vignettes in co-design workshops as part of the health literacy intervention development in this research project (following the OPHELIA framework 11,12), presents a potentially valuable way of enabling young people to communicate their understanding of and exploring their health concerns [4]. Following Ophelia, these structured co-design workshops will be used to engage key stakeholders (for example students, teachers, school management, families) to generate a broad range of ideas about what is needed to support school health literacy [47]. The vignettes that have been developed will allow for this next phase of work with the schools to have a very clear and targeted focus, with these contextually relevant vignettes acting as a starting point for participants to consider the challenges being faced in their school. The vignettes themselves are crucial in this, in that they will allow these stakeholders to talk freely, without judgement, and allow for the production of intervention ideas that will be relevant and meaningful to their context. Notably, this aligns with the approach advocated by the NCCA [44] for Irish schools, in their emphasis of the work of Noddings [48];

*‘It is important that, as educators, we do not assume that we know what children need and design the curriculum to satisfy these assumed needs. Students should have opportunities to express their needs so that wellbeing programmes are developed that respond to their real and expressed needs rather than adult perceptions of what they need’ (pp 28)*

If successful, this health literacy intervention work may offer a roadmap for other schools in Ireland to take a similar student-centred approach to meet the outlined guidelines of the NCCA [44].

The vignette validation process detailed in this study proved to be critically important. This final stage of what was already an iterative development process, highlighted key health topics that had not already been captured in the extensive qualitative and quantitative work that had been carried out – and resulted in the creation of a further (ninth) vignette. Issues that were unearthed in this final validation stage, highlight the nuanced challenges around being careful to not creating stereotypical and therefore unrelatable characterisations, and demonstrated the importance of language [4, 46]. The words and phrases used in the vignettes was particularly important to the young people in this study, who had a distinct use of language [46]. Using the participants own words from the focus groups proved to be an extremely powerful way to remain authentic and achieve engagement from the student cohort in particular in the process. This also extended to the naming of each of the vignette ‘characters’. We would therefore recommend future research should look to include participants own language and some form of validation process that continues to engage with the participants.

In previous research, the vignettes themselves have been presented in text form to participants [12]. Whilst the validation process identified that the students felt the length of the vignettes was appropriate, and Flesch-Kincaid reading scores confirmed them suitable for the projects age range, given the generally low levels of literacy in young people in this context [18], it is possible that written vignettes may still present a barrier for engagement. As a result, it may be necessary to consider alternative novel approaches to enable young people to connect with the vignettes, for example, this may include the use of images, audio and videos to supplement the text and improve accessibility and inclusivity.

## Conclusion

The current paper details part of Phase One of the IHF’s Schools Health Literacy project, which is a registered WHO National Health Literacy Demonstration Project, conducted in May 2019. It was deemed inappropriate to retrospectively frame this study with a COVID-19 narrative given all of the work, and indeed subsequent work employing these Vignettes to co-design intervention strategies with young people, was carried out prior to the pandemic. Nevertheless, this global pandemic will have huge implications, that are yet to be fully understood. The health literacy of young people will likely have renewed significance as the world enters the recovery stage, and the perceptions of young people regarding their health and health literacy remain invaluable. As a result, future work of the current project will require the series of vignettes to be re-introduced, explored and refined with stakeholders to investigate how COVID-19 has impacted the health and health literacy of young people in this context, and the extent to which the vignettes may now need to be refined.

The methodology undertaken presents a useful protocol for co-creating robustly, locally-developed vignettes that can be replicated by others interested in exploring and improving the health literacy and health outcomes in different contexts. In the present study, findings emphasise the number of health issues faced by young people, and the potential barriers to improve their health literacy and subsequent health outcomes. Following a co-design approach, the critical next step is to continue to work with participating schools to enable them to overcome these barriers and facilitate positive and sustainable culture change. The creation of a series of authentic, meaningful and engaging vignettes will be immediately impactful in this project as a way to engage participants in health literacy research, with the subsequent intervention positioned to support these young people in the wake of COVID-19.

## Supplementary Information

**Additional file 1: Supplementary File 1** Adapted Questionnaire.

**Additional file 2: Supplementary File 2** Focus Group Guide.

**Additional file 3: Supplementary File 3** Vignette examples.

## Data Availability

The datasets used and/or analysed during the current study are available from the corresponding author on reasonable request.

## Abbreviations

COVID-19: Coronavirus Disease 2019
IHF: Irish Heart Foundation
WHO: World Health Organisation
