# Supplementary material for: Understanding disadvantaged adolescents’ perception of health literacy through a systematic development of peer vignettes
Source: BMC Public Health. 2021 Mar 25;21:593. doi: 10.1186/s12889-021-10634-x (PMC7992854; doi:10.1186/s12889-021-10634-x)
Supplement: Supplementary file 1 — Additional file 1: Supplementary File 1 Adapted Questionnaire. [file 12889_2021_10634_MOESM1_ESM.docx]

**Title:** Understanding disadvantaged adolescents' perception of health literacy through a systematic development of peer vignettes

Hannah R Goss^1*^, Clare McDermott^2^, Laura Hickey^3^, Johann Issartel^1^, Sarah Meegan^1,^ Janis Morrissey^3^, Celine Murrin^4^, Cameron Peers^1^, Craig Smith^1^, Ailbhe Spillane^4^, Sarahjane Belton^1^

^1^School of Health and Human Performance, Dublin City University, Dublin, Ireland

^2^Department of Sport and Health, Athlone Institute of Technology, Westmeath, Ireland

^3^The Irish Heart Foundation, Dublin, Ireland

^4^School of Public Health Physiotherapy and Sports Science, University College Dublin, Dublin, Ireland

***Correspondence:**Hannah Goss
[Hannah.goss@dcu.ie](mailto:Hannah.goss@dcu.ie)

**Supplementary File 1 Adapted Questionnaire**

**Lifestyle behaviours**

| **Question** | **Response** | **Source** |
| --- | --- | --- |
| My lifestyle is usually | Very Unhealthy  Unhealthy  OK  Healthy  Very Healthy | Bay et al., New Zealand |
| My physical health is | Very Bad  Bad  OK  Good  Very Good | Bay et al., New Zealand |
| My own health is |  | Bay et al., New Zealand |
| My activity levels are usually | Very Unhealthy  Unhealthy  OK  Healthy  Very Healthy | Researcher devised |
| The food I eat is usually |  | Bay et al., New Zealand |
| Even if I am busy, I make time to be healthy | Strongly disagree  Disagree  Agree  Strongly agree | Health Literacy Questionnaire (HLQ) |
| My screen time is usually | Very Unhealthy  Unhealthy  OK  Healthy  Very Healthy | Researcher devised |
| My mental health is | Very Bad  Bad  OK  Good  Very Good | Researcher devised |
| There are things I do regularly to make myself more healthy | No  Yes | HLQ |

**Info on risky behaviors**

| **Question** | **Response** |  |
| --- | --- | --- |
| I think I have good information about the impact of drinking alcohol on my health | Strongly disagree  Disagree  Agree  Strongly Agree | Researcher devised |
| I think I have good information about the impact of smoking cigarettes on my health |  | Researcher devised |
| I think I have good information about the impact of drugs on my health |  | Researcher devised |
| I think I have good information about the impact of vaping on my health |  | Researcher devised |
| I think I have good information about the impact of screen time on my health |  | Researcher devised |

**Info on positive healthy behaviors**

| **Question** | **Response** |  |
| --- | --- | --- |
| I have all the information I need to look after my health | Strongly disagree  Disagree  Agree  Strongly Agree | HLQ |
| I think I have good information about the impact of food on my health |  | Researcher devised |
| I have good information about health |  | HLQ |
| I think I have good information about the impact of physical activity on my health |  | Researcher devised |
| I think I have good information about the impact of sleep on my health |  | Researcher devised |

**Health information from media sources**

| **Question** | **Response** |  |
| --- | --- | --- |
| I get health information from newspapers or magazines | Never  Sometimes  Always | Researcher devised |
| I get health information from the radio |  |  |
| I get health information from sponsored content online |  |  |
| I get health information from advertisements |  |  |
| I get health information from TV |  |  |
| I get health information from Snapchat |  |  |
| I get health information from YouTube |  |  |
| I get health information from Google |  |  |
| I get health information from Netflix/live streaming |  |  |
| I get health information from Instagram |  |  |

**Social support**

| **Question** | **Response** |  |
| --- | --- | --- |
| I have a lot of support from my friends to do healthy things | Strongly disagree  Disagree  Agree  Strongly Agree | HLQ |
| When I don’t feel great I have people around me that really understand what I am going through |  | HLQ |
| I get health information from my friends | Never  Sometimes  Always | Researcher devised |
| I have a lot of support from my family to do healthy things | Strongly disagree  Disagree  Agree  Strongly Agree | HLQ |
| I get health information from my parents/ guardians | Never  Sometimes  Always | Researcher devised |

**Understanding health information**

| **Question** | **Response** |  |
| --- | --- | --- |
| Understanding health information I see in pictures is | Always difficult  Usually difficult  Sometimes difficult  Usually easy  Always easy | Researcher devised |
| Understanding health information I see in videos is |  | Researcher devised |
| Reading and understanding written health information is |  | HLQ |

**Effect of lifestyle on health**

| **Question** | **Response** |  |
| --- | --- | --- |
| The physical activity that I do now will affect my future | I don’t know  Strongly disagree  Disagree  Agree  Strongly Agree | Researcher devised |
| The food that I eat now will affect my health in the future |  | Bay et al., New Zealand |
| It is important for me to eat healthy food now |  | Bay et al., New Zealand |
| How much does it matter what you eat | Not at all  Not very much  Quite a lot  A lot | Bay et al., New Zealand |
| How much does it matter whether or not you are healthy |  | Bay et al., New Zealand |
| How much does it matter whether or not you are active or exercise every day |  | Bay et al., New Zealand |

**Appraisal of health information**

| **Question** | **Response** |  |
| --- | --- | --- |
| When I get information from different places I compare them to see which is better | Never  Sometimes  Always | HLQ |
| When I see new information about health I check to see if it is true or not | Strongly disagree  Disagree  Agree  Strongly Agree | HLQ |
| I get information from healthcare professionals | Never  Sometimes  Always | Researcher devised |
