# Supplementary material for: Understanding disadvantaged adolescents’ perception of health literacy through a systematic development of peer vignettes
Source: BMC Public Health. 2021 Mar 25;21:593. doi: 10.1186/s12889-021-10634-x (PMC7992854; doi:10.1186/s12889-021-10634-x)
Supplement: Supplementary file 2 — Additional file 2: Supplementary File 2 Focus Group Guide. [file 12889_2021_10634_MOESM2_ESM.docx]

**Title:** Understanding disadvantaged adolescents' perception of health literacy through a systematic development of peer vignettes

Hannah R Goss^1*^, Clare McDermott^2^, Laura Hickey^3^, Johann Issartel^1^, Sarah Meegan^1,^ Janis Morrissey^3^, Celine Murrin^4^, Cameron Peers^1^, Craig Smith^1^, Ailbhe Spillane^4^, Sarahjane Belton^1^

^1^School of Health and Human Performance, Dublin City University, Dublin, Ireland

^2^Department of Sport and Health, Athlone Institute of Technology, Westmeath, Ireland

^3^The Irish Heart Foundation, Dublin, Ireland

^4^School of Public Health Physiotherapy and Sports Science, University College Dublin, Dublin, Ireland

***Correspondence:**Hannah Goss
[Hannah.goss@dcu.ie](mailto:Hannah.goss@dcu.ie)

**Supplementary File 2 Focus Group Guide**

**Questions:**

Card sorting activity 1: Get participants to choose the words from above (participants provided with card examples of food/smoking/drinking/going to the doctors/physical activity/mental health/wellbeing/screen time/sleep/going to the dentist/hygiene) that they think are important for their health and those that they consider are unhealthy.

What are the top 4 and why?

Do you think we have left out any words?

Are there other things that are important?

Divide the pictures into what you think represent healthy and unhealthy

Prompt discussion on what it means to be healthy with reference to the pictures/prompts

What do you think are the most common unhealthy behaviors kids your age take part in?

Why do you think adolescents do this?

Prompt discussion by referring to the picture cards from activity

2. Who do you think is in charge/responsible for your health?

Why do you think this?

Prompt discussion with School, community, environment, parents, peers

3. Do you think people your age get information about health? and if so where do you think they get it?

Prompt discussion by referring to the picture cards from Q1

Where have you ever come across information on health?

Do you ever see pictures/videos/written information about healthy or unhealthy behaviors? Where do you see this?

Prompt discussion with TV, parents, social media, peers

Card sorting activity 2: Give students three picture cards each and ask ‘Write down your top three places /people that you would look for this information’. Get participants to place the cards on the table. Get participants to arrange in order of which sources they would trust the most.

Why do you think they are the most trustworthy?

Why would you think the ones you have listed at the bottom are less accurate?
