# Supplementary material for: Understanding disadvantaged adolescents’ perception of health literacy through a systematic development of peer vignettes
Source: BMC Public Health. 2021 Mar 25;21:593. doi: 10.1186/s12889-021-10634-x (PMC7992854; doi:10.1186/s12889-021-10634-x)
Supplement: Supplementary file 3 — Additional file 3: Supplementary File 3 Vignette examples. [file 12889_2021_10634_MOESM3_ESM.docx]

**Title:** Understanding disadvantaged adolescents' perception of health literacy through a systematic development of peer vignettes

Hannah R Goss^1*^, Clare McDermott^2^, Laura Hickey^3^, Johann Issartel^1^, Sarah Meegan^1,^ Janis Morrissey^3^, Celine Murrin^4^, Cameron Peers^1^, Craig Smith^1^, Ailbhe Spillane^4^, Sarahjane Belton^1^

^1^School of Health and Human Performance, Dublin City University, Dublin, Ireland

^2^Department of Sport and Health, Athlone Institute of Technology, Westmeath, Ireland

^3^The Irish Heart Foundation, Dublin, Ireland

^4^School of Public Health Physiotherapy and Sports Science, University College Dublin, Dublin, Ireland

***Correspondence:**Hannah Goss
[Hannah.goss@dcu.ie](mailto:Hannah.goss@dcu.ie)

**Supplementary File 3 Vignette examples**

| Vignette 1. ‘John’ | | | | | | | |
| --- | --- | --- | --- | --- | --- | --- | --- |
| Lifestyle behaviours | Information on risky behaviours | Information on positive behaviours | Health Information from media | Social support | Understanding health information | Effect of lifestyle on health | Appraisal of health information |
| 7.15 | 3.59 | 3.75 | 4.52 | 3.96 | 2.23 | 5.24 | 1.76 |
| *Brief Descriptive overview from cluster analysis:*  Cluster 1 accounted for the largest proportion of the sample including 201 participants (21%), with a large majority of boys, n=134 compared with girls, n= 67. Students in this cluster scored high in lifestyle behaviours, social support and effect of lifestyle on health. Students in this group scored low on the Information on risky behaviours and health Information from media, potentially meaning that the feel they don’t have enough Information and that they Information they do attain is not from media sources. Physical activity levels for participants this cluster were second highest of all clusters with students reporting to be physically active for at least 60 minutes on 4.59 days per week. | | | | | | | |
| *Final Vignette (derived from qualitative data):*  John is 12. He loves football. He has training twice a week and a match every weekend. All of his friends play football too, so it’s a good way for him to hang out with them. The only other place that lets him and his mates hang out is McDonald’s, they usually go there after school. He worries he shouldn’t be eating junk food every day, but John sees a lot of ads for McDonald’s and KFC, and it always makes him hungry. John doesn’t really believe any of the health stuff he sees on social media because you can never know if it’s true or not.  None of John’s friend’s smoke or take drugs or anything like that, but he knows there’s a lot of lads at school that do. As he’s got older, he knows he has more responsibility to make healthy choices, but his parents still have a big impact. His mam’s the one that does the food shop, and she’s always trying to get him to eat different foods, and his Dad’s the one that drops him to football.  At school, John’s favourite classes are PE and SPHE. Some of the other teacher’s he has stress him out, but the PE and SPHE teachers are really sound. He knows they’ve have been on courses to learn things about mental health and healthy eating. But he’s worried that his PE and SPHE lessons are going to change after third year, with exams and stuff. | | | | | | | |

| Vignette 2a. ‘Amy’ | | | | | | | |
| --- | --- | --- | --- | --- | --- | --- | --- |
| Lifestyle behaviours | Information on risky behaviours | Information on positive behaviours | Health Information from media | Social support | Understanding health information | Effect of lifestyle on health | Appraisal of health information |
| 7.49 | 4.46 | 4.44 | 5.66 | 4.24 | 2.51 | 5.29 | 2.28 |
| *Brief Descriptive overview from cluster analysis:*  Cluster 2 represented 120 students, 13% of the sample. These students scored highest for all factors however not all significantly different from other clusters. Participants in Cluster 2 had significantly higher scores than all other variables for lifestyle behaviours, Information on positive behaviours, social support and appraisal of health Information. Students in cluster 2 also reported the highest physical activity levels of all clusters, reporting to be physically active for at least 60 minutes on 4.7 days of a typical week. This was significantly higher than all other clusters with the exception of cluster one. | | | | | | | |
| *Final Vignette (derived from qualitative data):*  Amy is 13. She loves keeping fit and plays lots of sports. She plays football twice a week and basketball on Wednesday. Amy feels it’s something to do, because if she didn’t do sports, she’d just be stuck at home. She knows that she’d just end up on her phone scrolling through the same stuff, and she’d just be bored out of her mind.  She’s started to go to the gym in her spare time and tries to eat healthily because she knows it’ll help her get better at her sport. She follows a few people on Instagram that give her tips to on how to get fitter and what foods to eat before matches. She didn’t know if she could trust the websites at first, but then she found out that it was all researched and tested by scientists, so it’s obviously proper Information.  Amy doesn’t drink or smoke, even though she knows some people at school do. It’s not like she’s trying to be an angel, but her and her friends try to stay away from stuff like that. They’ve been told in school how bad it is for you.  Since Amy’s mam and dad separated, Amy’s mam takes her to all of training and matches. It’s been weird because her Dad always used to take her, and they used to talk about football together. Her mam’s really supportive of all her sport. Even though has to rush home from work, she always makes sure dinner is ready when she gets in from school and has healthy snacks ready for her after training. Amy’s still found her ma and da separating hard but lately she’s been upset. She doesn’t talk about it with her parents because it’ll make them feel bad, but her friend Sarah gets it because her parents are separated too. Talking about it with Sarah makes her feel better. | | | | | | | |

| Vignette 2b. ‘Ryan’ | | | | | | | |
| --- | --- | --- | --- | --- | --- | --- | --- |
| Lifestyle behaviours | Information on risky behaviours | Information on positive behaviours | Health Information from media | Social support | Understanding health information | Effect of lifestyle on health | Appraisal of health information |
| 7.49 | 4.46 | 4.44 | 5.66 | 4.24 | 2.51 | 5.29 | 2.28 |
| *Brief Descriptive overview from cluster analysis:*  Cluster 2 represented 120 students, 13% of the sample. These students scored highest for all factors however not all significantly different from other clusters. Participants in Cluster 2 had significantly higher scores than all other variables for lifestyle behaviours, Information on positive behaviours, social support and appraisal of health Information. Students in cluster 2 also reported the highest physical activity levels of all clusters, reporting to be physically active for at least 60 minutes on 4.7 days of a typical week. This was significantly higher than all other clusters with the exception of cluster one. | | | | | | | |
| *Final Vignette (derived from qualitative data):*  Ryan is 14. Ryan plays football, hurling and soccer for his local clubs. He has been called for trials for the u15 Dublin GAA development squad. It’s been his dream to play for Dublin since he was a kid, just like his Dad. They train twice a week and have separate gym and recovery sessions. He loves it, but it can be pretty exhausting. His two friends are on the development squad as well so they are all working as hard as they can, so they can stay on the team. Ryan is struggling in school because he wants to be the perfect athlete. His grades aren’t great and he’s not interested in school. But the teachers make it stressful. He doesn’t get a lot of time to do homework, and he only gets 6 hours sleep a night. He usually goes to bed 12 to 1 ish and is up at 6 or so. He knows a lot of his friends smoke but he knows he’d lose his place on the squad if he did.  Even though his Mam and Dad aren’t together anymore, his Dad still comes to all his matches, and he knows both his parents are really proud of him for getting trials for the squad. His Mam pays for his gym membership, and him and his mates go there together because they’re all trying to get bigger and stronger. He told his Dad, so he’s started showing him different exercises to try out in the gym. Sometimes this can be a bit much. Ryan follows all of the Dublin players on Instagram and they’re always posting videos of them in the gym and all the food they eat. He tries to copy what they do because he wants to be as good as them. One of the players on Instagram said it was really important to eat protein straight after a session, so sometimes he cooks some of his own extra food for after the gym, but usually he just has a protein shake. | | | | | | | |

| Vignette 4a. ‘Lauren’ | | | | | | | |
| --- | --- | --- | --- | --- | --- | --- | --- |
| Lifestyle behaviours | Information on risky behaviours | Information on positive behaviours | Health Information from media | Social support | Understanding health information | Effect of lifestyle on health | Appraisal of health information |
| 5.12 | 3.14 | 2.91 | 4.35 | 2.85 | 1.64 | 3.76 | 1.32 |
| *Brief Descriptive overview from cluster analysis:*  Cluster 4 represents 96 students, 10% of the sample. The participants in this group had the lowest scores for all factors with the exception of Information from media sources. Scores from lifestyle behaviours, Information on positive behaviours, social support, understanding health Information, effect of lifestyle on health and appraisal of health Information were significantly lower than all other clusters. Students in cluster 4 also reported the lowest physical activity levels of all clusters, reporting to be physically active for at least 60 minutes on 2.7 days of a typical week. This was significantly lower than all other clusters with the exception of cluster three. | | | | | | | |
| *Final Vignette (derived from qualitative data):*  Lauren is 15 years old. Lauren can see how bad drink and drugs are for you because she has seen how it has changed her Mam. This has affected her family a lot. Lauren sometimes has to cook for her and her brother as her Mam goes out a lot in the evenings with her friends and her Dad’s at work. Their favourite thing is pizza because it’s easy and doesn’t take too long to cook. She doesn’t really know how to cook a proper meal, even though she’d like to try and make some of the stuff she’s seen on Instagram.  Lauren loves Instagram and she’s always on her phone. She loves looking at stories where she can see people doing exercises and talking about what you should eat to get toned. Sometimes Lauren is just scrolling through Instagram and she’ll see a post about how to get your dream body. She likes to try some of these diets, especially before a big night out. Lauren says it’s okay because she only does it for the week before the night out and not all the time like other girls. The week after she can go back to eating chocolate and crisps- they’re her favourite. She can be a bit self-conscious about what she looks like. All of her friends tell her she’s the smallest, but she doesn’t believe them. She’s thinking of maybe not eating for the week before her next night out so she can lose a bit more weight.  When they go on nights out, one of her friends will try to take some drink from their mam’s drinks cabinet and they share it out between them. Lauren and her friends love doing this because it gets them in the mood for the night out. Her and her friends stopped smoking though. Now they vape because it’s cool. One of her friend’s friends sells it, and they can get nicer flavours and it doesn’t smell like normal smoking. | | | | | | | |

| Vignette 4b. ‘Luke’ | | | | | | | |
| --- | --- | --- | --- | --- | --- | --- | --- |
| Lifestyle behaviours | Information on risky behaviours | Information on positive behaviours | Health Information from media | Social support | Understanding health information | Effect of lifestyle on health | Appraisal of health information |
| 5.12 | 3.14 | 2.91 | 4.35 | 2.85 | 1.64 | 3.76 | 1.32 |
| *Brief Descriptive overview from cluster analysis:*  Cluster 4 represents 96 students, 10% of the sample. The participants in this group had the lowest scores for all factors with the exception of Information from media sources. Scores from lifestyle behaviours, Information on positive behaviours, social support, understanding health Information, effect of lifestyle on health and appraisal of health Information were significantly lower than all other clusters. Students in cluster 4 also reported the lowest physical activity levels of all clusters, reporting to be physically active for at least 60 minutes on 2.7 days of a typical week. This was significantly lower than all other clusters with the exception of cluster three. | | | | | | | |
| *Final Vignette (derived from qualitative data):*  Luke is 15. Luke has been told in school that smoking is bad for you, but he still does it. All his family smoke, and he and his mates started last year. Smoking helps him relax when he’s stressed. His teachers are always annoying him about homework and exams. He feels that smoking lets him turn off for like an hour and he can forget about everything that’s happening at school.  Luke hates when adults try and tell him about being healthy and not smoking or drinking. He thinks what the teachers says doesn’t really matter because they don’t really care about him. Luke thinks the teachers don’t know anything. Compared to him, they’ve had an easy life. The people that he follows on Instagram, they understand him more. They get what he’s going through because they grew up in a similar area. Luke can’t wait until he can leave school and go off and get a job. He says the school just repeat the same thing over and over again, so it’s not actually teaching you anything, they just want to get you through the exams.  Sometimes when Luke and his friends hang out in the park, fights break out between different groups. It’s hard to avoid getting involved because Luke’s big so people always start on him, and he can’t back out. The other day in the park some lad tried to sell Luke and his friends some weed. He said no, so his friends laughed at him. He’s heard about people smoking weed when they’re sick and he see’s loads of stuff on Instagram, so it can’t be that bad. Next time it’ll just be easier to join in rather than being laughed at again. | | | | | | | |

| Vignette 5. ‘Dylan’ | | | | | | | |
| --- | --- | --- | --- | --- | --- | --- | --- |
| Lifestyle behaviours | Information on risky behaviours | Information on positive behaviours | Health Information from media | Social support | Understanding health information | Effect of lifestyle on health | Appraisal of health information |
| 6.07 | 3.66 | 3.48 | 4.08 | 3.37 | 2.14 | 4.25 | 1.47 |
| *Brief Descriptive overview from cluster analysis:*  Cluster 5 accounted for 20% of the study group with a greater proportion of male to female (male n = 109, female n = 79). For Information from media sources, this cluster had the lowest score. All of the factors in this cluster were on the negative end of the spectrum however not as low as cluster 4. Participants in this cluster were physically active for an average of 60 minutes on 3.67 days per week. | | | | | | | |
| *Final Vignette (derived from qualitative data):*  Dylan is 13. Dylan’s favourite thing to do is play on his PlayStation. He’s very quiet and doesn’t have that many friends at school. But when he’s online, he can play against all his friends on FIFA, Call of Duty or Fortnite. Dylan finds the games addictive, and he normally comes straight in from school and plays all evening. When he’s at his Mam’s, she makes him take a break to eat dinner, but then goes straight back online. Dylan usually stays up fairly late playing Playstation with his friends and goes to bed between 12 and 3am. Sometimes he gets tired while playing, but he just has a can of monster to keep him going. He’s usually pretty tired when he has to get up in the morning, and sometimes he’s late to school because he was up so late the night before. He never has time to do his homework either, so his teachers have started giving out about that too.  Dylan knows that sitting in all weekend is bad for him but still it’s better than him going out and drinking and smoking. When he’s at his Mam’s, she makes him go out he to meet the lads. They usually just go hang out in McDonald’s. Dylan always gets a coke and a double cheeseburger and then they can just stay in there for the afternoon. But sometimes they go down the park. Dylan doesn’t really like doing this because there’s always fights or cars being robbed. Where he lives, there are always gangs hanging out in the park just looking for trouble. Dylan says that if you’re on your own walking up the road they shout at you and follow you, and he just tries to avoid them. It puts him off going out with his friends. It’s just easier to sit in and play on his PlayStation. | | | | | | | |

| Vignette 6. Jay | | | | | | | |
| --- | --- | --- | --- | --- | --- | --- | --- |
| Lifestyle behaviours | Information on risky behaviours | Information on positive behaviours | Health Information from media | Social support | Understanding health information | Effect of lifestyle on health | Appraisal of health information |
| 6.32 | 4.41 | 4.09 | 4.36 | 3.51 | 2.48 | 4.96 | 2.06 |
| *Brief Descriptive overview from cluster analysis:*  Cluster 6 accounted for 16% of the study sample, with 155 students. There was twice the number of male to female students in this group. Students in this cluster scored second highest for Information on risky behaviours, Information on positive behaviours as well as appraisal of health Information and second lowest on health Information from media sources. This potentially means that students in this cluster felt they had a lot of Information on health behaviours and their ability to appraise them and again did not source their Information from the media. The lack of social support in this group may potentially mean that this group was more independent. Students in this cluster were physically active for 60 minutes on 3.81 days per week. | | | | | | | |
| *Final Vignette (derived from qualitative data):*  Jay is 15. He plays football, but he doesn’t like it, so he’s thinking of stopping. He’d rather join the gym like his brother and the lads he follows on Instagram, but he can’t afford it and his parents won’t give him the money. They say it’s not worth it, and his mam says he shouldn’t be going to the gym while he’s still growing. But they haven’t got a clue about anything. So, he just uses the gym in school, but he can only use it at certain times and it’s not as good as the one his brother goes too.  He never runs in the gym or anything like that, he just does weights. Jay takes pictures of himself in the gym and posts them on snapchat and Instagram. Even if he’s not in the gym, he takes pictures and posts them. Jay follows big body builders and sports people on social media and tries to copy them.  He always makes sure he eats loads of protein because he says you need it to get big. For lunch, he grabs 2 chicken fillet rolls in the school canteen. He’s heard of some other lads at school taking extra supplements to help them get bigger and lift heavier weights, but he doesn’t know what’s in them, so he’s not sure. | | | | | | | |

| Vignette 7. ‘Sarah’ | | | | | | | |
| --- | --- | --- | --- | --- | --- | --- | --- |
| Lifestyle behaviours | Information on risky behaviours | Information on positive behaviours | Health Information from media | Social support | Understanding health information | Effect of lifestyle on health | Appraisal of health information |
| *Brief Descriptive overview from cluster analysis:*  This additional profile was developed after feedback from students and teachers during the validation phase who felt ‘someone was missing’. Stakeholders felt this person was typically female, who used to regularly participate in physical activity, but these levels have declined. Scores across factors would be above average, with Information from media sources and Information on risky behaviours would be amongst the highest across the vignettes. This description came from a validation session with teachers:  *“(She) Loses confidence in wearing any kind of pe gear and also in physical activity and is more and more conscious about their weight.*  *On social media a lot and sees a lot of women toning and putting up inspirational stories and videos that they find intimidating*  *Used to walk a lot in the evening with friends but getting used to staying in as they feel unsafe heading out especially towards darkness.”* | | | | | | | |
| *Final Vignette (derived from qualitative data):*  Sarah is 12. Sarah’s shy, but she has a close group of friends she hangs round with. She always gets her homework done and does pretty well in school. Sarah doesn’t mind PE, but she definitely doesn’t like it as much as other classes. She doesn’t really like using the changing rooms.  She doesn’t play sport or anything like that outside of PE, but neither do her sisters. Sometimes Sarah will take the dog out for a walk, but that’s the most she’ll do. Sarah thinks she’s a bit overweight, but it’s ok, because all her family and friends are too. She thinks it’s just normal and she’s happy most of the time, apart from non-uniform days. She hates non-uniform days and having to come in her own clothes. Sarah doesn’t like shopping or wearing the clothes the other girls at school like. She doesn’t think they suit her and she feels self-conscious. So on non-uniform days she’ll say she’s sick, and her Ma lets her stay home.  Her Mam loves cooking, and always makes sure Sarah and her sisters finish everything on their plates, even if they’re not that hungry. If she tries to leave anything, her Mam always gives out about her wasting stuff. In the evenings, Sarah sits in and watches telly, she loves the soaps, and she always has some chocolate to snack on. | | | | | | | |
